# Maternal SARS-CoV-2 Vaccination and Infant Protection Against SARS-CoV-2 During the First 6 Months of Life

**DOI:** 10.21203/rs.3.rs-2143552/v1

**Published:** 2022-10-18

**Authors:** Ousseny Zerbo, G. Thomas Ray, Bruce Fireman, Evan Layefsky, Kristin Goddard, Edwin Lewis, Pat Ross, Saad Omer, Mara Greenberg, Nicola Klein

**Affiliations:** Kaiser Permanente Northern California, Vaccine Study Center; Kaiser Permanente Northern California, Vaccine Study Center; Kaiser Permanente Northern California, Vaccine Study Center; Kaiser Permanente Northern California, Vaccine Study Center; Kaiser Permanente Northern California, Vaccine Study Center; Kaiser Permanente Northern California, Vaccine Study Center; Kaiser Permanente Northern California, Vaccine Study Center; Yale University; Obstetrics and Gynecology, Kaiser Permanente Northern California Oakland, Regional Perinatal Service Center, Kaiser Permanente Northern California, Santa Clara; Kaiser Permanente Northern California, Vaccine Study Center

## Abstract

We examined the effectiveness of maternal vaccination against SARS-CoV-2 infection in 30,288 infants born at Kaiser Permanente Northern California from December 15, 2020, to May 31, 2022. Using Cox regression, the effectiveness of maternal vaccination was 85% (95% confidence interval [CI]: 67, 93), 64% (CI: 43, 78) and 57% (CI: 36,71) during the first 2, 4 and 6 months of life, respectively, in the Delta variant period. In the Omicron variant period, the effectiveness of maternal vaccination in these three age intervals was 22% (CI: −18,48), 14% (CI: −10,32) and 12% (CI: −4,26), respectively. Over the entire study period, the incidence of hospitalization for COVID-19 was lower during the first 6 months of life among infants of vaccinated mothers compared with infants of unvaccinated mothers (21/100,000 person-years vs. 100/100,000 person-years). Maternal vaccination was protective, but protection was lower during Omicron than during Delta. Protection during both periods decreased as infants aged.

## Introduction

In the US, as of the end of September 2022, almost 15 million children have tested positive for severe acute respiratory syndrome corona virus 2 (SARS-CoV-2), the virus that causes Coronavirus Disease 2019 (COVID-19). Children currently account for about 18.5% of reported COVID-19 cases in the US^1^. SARS-CoV-2 infection can lead to severe illnesses and hospitalizations in children and infants^2–5^. During Omicron predominance, children aged <6 months accounted for 44% of hospitalizations among children ages 0 – 4 years^3^.

Vaccination offers the best way to protect against COVID-19 and its complications. COVID-19 vaccines have demonstrated both high efficacy in clinical trials and high real-world effectiveness, especially against the original and Delta variant of the virus^6–10^. Real-world data suggest lower COVID-19 vaccine effectiveness against Omicron variants^11–14^. However, infants aged < 6 months are not currently eligible for any currently available COVID-19 vaccines and must rely on placentally acquired immunity from their mothers.

Like influenza and Tdap vaccines^15,16^, data suggest that vaccination during pregnancy may protect infants who are not old enough to be vaccinated against COVID-19. Two recent epidemiological studies found that vaccination during pregnancy was associated with a reduced risk of SARS-CoV-2 infection in infants during their first 4 months of life and a reduced risk of hospitalization during the first 5 months of life^17,18^.

The objective of this study was to further evaluate the effectiveness of at least 2 doses of mRNA COVID-19 vaccination during pregnancy for preventing SARS-CoV-2 infection in infants during the first 2, 4 and 6 months of life during the Delta and Omicron variant periods. We used two different study designs: a primary design using a cohort analysis in which infants of vaccinated pregnant persons were compared with infants of unvaccinated pregnant persons. In this design, we used Cox proportional hazards models with calendar days as the underlying scale to estimate hazard ratios and calculated vaccine effectiveness as 1 minus the hazard ratio. Secondarily, we used a Test Negative Design (TND), which is a case control study, to compare the odds of vaccination among mothers of infants who tested positive vs. the odds of vaccination among mothers of infants who tested negative. In this analysis, vaccine effectiveness was evaluated as 1 minus the odds ratio. The aim of the secondary design was to compare the results from the cohort and TND design.

## Results

### Descriptive statistics and characteristics

**Figure 1:**
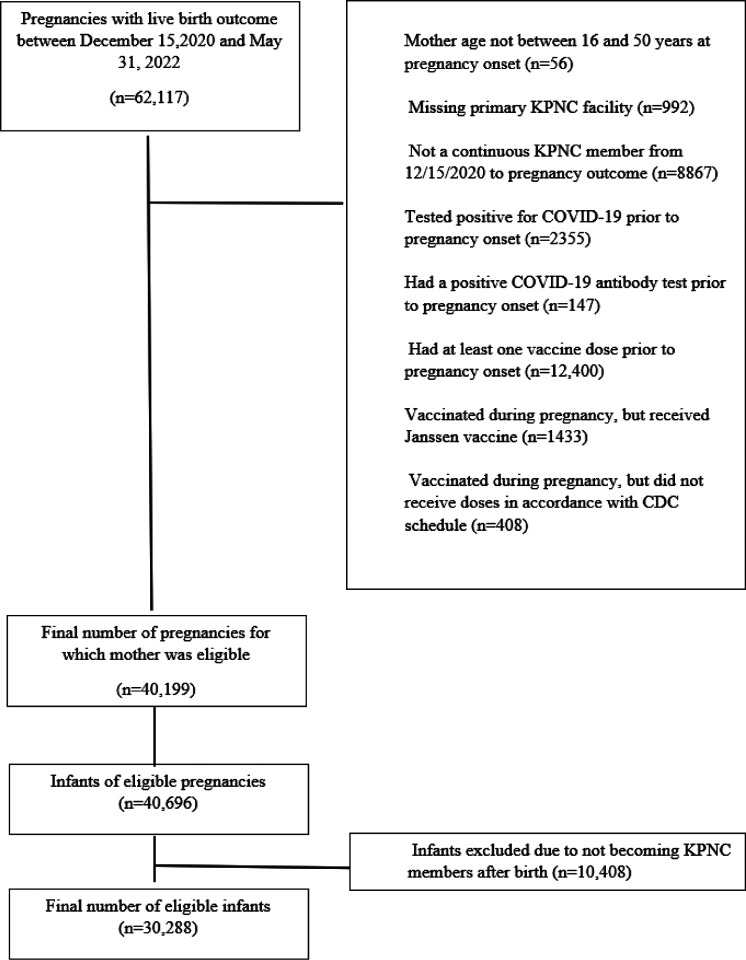
Construction of study cohort. Children born at Kaiser Permanente Northern California from December 15, 2020, through May 31, 2022.

Between December 15, 2020, and May 31,2022, we identified 62117 infants born at Kaiser Permanente Northern California (KPNC), an integrated health care delivery organization. Among these infants, we excluded 21918 (35.3%) based on maternal exclusion criteria and 10408 (16.0%) after applying infant exclusion criteria (Figure 1). The final study population included 30288 (48.8%) infants who were KPNC members at least 2 months after birth. The mean age at pregnancy onset was 31 years (standard deviation 4.66 years). Most mothers (66.15%) were between ages 25 and <35 years, and more than a quarter (27.26%) were of Asian race, 5.16% were Black, 24.45% were of Hispanic ethnicity and 37.57% were White. Among the infants in the cohort, 19179 (63.32%) of the mothers were unvaccinated during pregnancy, 1035 (3.42%) of the mothers received 1 dose of a mRNA COVID-19 vaccine, 9456 (31.22%) received 2 doses, and 618 (2.04%) received 3 doses during pregnancy (Table 1).

During the first 6 months of life, 940 (3.10%) infants tested positive for SARS-CoV-2 by polymerase chain reaction (PCR) test and 10 (0.03%) infants were hospitalized with a positive SARS-CoV-2 test.

### Vaccine effectiveness: Primary design cohort analyses

During the Delta dominant period, the crude incidences of testing positive for SARS-CoV-2 during the first 2, 4 and 6 months of life were lower among infants whose mothers received at least 2 doses of mRNA COVID-19 vaccines during pregnancy (0.74, 1.38, and 1.55 infants per 100 person years [PY], respectively) than those whose mothers were not vaccinated during pregnancy (5.53, 5.18, and 4.83 infants per 100 PYs, respectively). After adjusting for covariates, vaccination during pregnancy significantly reduced risk of the infant testing SARS-CoV-2 positive by 85% (95% confidence interval [CI]: 67, 93) during the first 2 months of life, 64% (95% CI: 43, 78) during the first 4 months of life and 57% (95% CI: 36,71) during the first 6 months of life (Table 2). During the Omicron dominant period, vaccination during pregnancy reduced the risk of the infant testing SARS-CoV-2 positive by 22% (95% CI: −18, 48) during the first 2 months of life, 14% (95% CI: −10, 32) during the first 4 months of life, and 12% (95% CI: − 4, 26) during the first 6 months of life (Table 2).

In supplemental analyses by trimester of vaccination, receipt of the second dose during the second and third trimester reduced the risk of infants testing SARS-CoV-2 positive during the Delta dominant period by 91% (95% CI: 63, 98) and 87% (95% CI: 55, 96), respectively, during the first 2 months of life, by 60% (95% CI: 23, 79) and 70% (95% CI: 43, 84) during the first 4 months of life and by 65% (95% CI:33, 81) and 54% (95% CI: 27, 71) during the first 6 months of life (Table 3). We observed a similar pattern in vaccine effectiveness by trimester during the Omicron dominant period, however, estimates of vaccine effectiveness by trimester during the Omicron period were imprecise and much lower than during the Delta period (Table 3).

Over the entire study period, the crude rate of hospitalization with a SARS-CoV-2 positive test was lower during the first 6 months of life among infants whose mothers received at least 2 doses of mRNA COVID-19 vaccines during pregnancy compared with infants whose mothers were unvaccinated during pregnancy (21/100000 PY vs. 100/100000 PY). VE against hospitalization was not estimated because of the very small number of hospitalized cases. There were only 1 hospitalized case among the children of vaccinated mothers and 9 hospitalized cases among the children of unvaccinated mothers (Table 1).

### Secondary analysis results using a test negative design (TND)

In the TND, we estimated that during the Delta predominant period, maternal vaccination reduced the infant’s risk of testing SARS-CoV-2 positive by 98% (95% CI:75, 100) during the first 2 months of life, 72% (95% CI: 55, 83) during the first 4 months of life, and 63% (95% CI: 45, 75) during the first 6 months of life (Supplemental Table). During the Omicron dominant period, maternal vaccination reduced the infant’s risk of testing SARS-CoV-2 positive by 55% (95% CI: 15, 76) during the first 2 months of life, 37% (95% CI:12, 55) during the first 4 months of life, and 40% (95% CI: 25, 53) during the first 6 months of life (Supplemental Table).

## Discussion

In this large study which included >30,000 infants, we found that receipt of at least 2 doses of mRNA COVID-19 vaccine during pregnancy was associated with a decreased risk of infants testing SARS-CoV-2 positive during their first 6 months of life. Maternal vaccination reduced the infant’s risk of testing SARS-CoV-2 positive initially by 85% which decreased to 57% by 6 months of life in the Delta dominant period. However, vaccination during pregnancy was less effective at protecting infants against SARS-CoV-2 infection during the Omicron period. As infants aged, protection provided by maternal vaccination decreased during both periods.

Although the study was unable to directly estimate VE against hospitalization due to the small number of hospitalized cases, it found that over the entire study period, the incidence rate of hospitalization during the first 6 months of life was much lower among the infants whose mothers were vaccinated during pregnancy compared with those whose mothers were not vaccinated. These results suggest that in addition to providing protection against testing positive, vaccination during pregnancy may also provide protection against hospitalization (severe disease) in the infants during their first 6 months of life as previously reported recently^18^.

Our findings that vaccination during pregnancy was effective at protecting infants during the Delta period are similar to those reported in a recent Norwegian study showing that mRNA COVID-19 vaccination during pregnancy was associated with a 71% decreased risk of testing positive for SARS-CoV-2 in infants during their first 4 months of life during the Delta period^17^. During the Delta period, we found that protection extended through the infant’s first 6 months of life. However, in contrast with the Norwegian study which reported that infants of mothers who were vaccinated had a 33% decreased risk of testing positive during the first 4 months of life during the Omicron period^17^, our study found a 14% reduced risk that was not statistically significant. Difference between the two studies might be due to population characteristics and to the timing of follow up as ours went through May 31, 2022, while the Norwegian study’s ended in April 2022.

The finding that maternal vaccination was less effective at protecting infants during the Omicron dominant period is also consistent with previous studies which have reported decreased effectiveness of mRNA COVID-19 vaccines during Omicron among children and adults^14,19^. Recently another study reported that the effectiveness of mRNA COVID-19 vaccines against infections and hospitalizations among pregnant people was higher during the Delta period than during the Omicron period^20^.

We observed that infant’s protection through vaccination during pregnancy decreased as they aged from 2 months to 6 months. These findings are consistent with diminishing of pregnancy derived antibodies in the infants over time^21^. A recent study found that the mean titer of maternally derived antibodies in infants of vaccinated mothers were higher at ages 2 months compared with antibody titers at ages 6 months^22^.

Despite several studies showing that vaccination during pregnancy is safe for pregnant people^11–15^, vaccine uptake has been suboptimal in this group^23^. In the present study, the mothers of only 33% of infants in the cohort received at least 2 doses. More efforts are needed to promote COVID-19 vaccines for pregnant persons because vaccination provides protection to mothers and their infants until they are old enough to receive their own COVID-19 vaccines.

Our study was strengthened both by its large sample size and our ability to follow infants through 6 months of age. In addition, our study period included two different SARV-CoV-2 variants, which allowed estimation of the effectiveness of vaccination during pregnancy in infants during both the Delta and Omicron variant periods. Our primary cohort analysis used calendar days as the underlying scale to ensure that we compared infants of vaccinated and unvaccinated mothers on the same calendar days because vaccination status during pregnancy and risk of SARS-CoV-2 infection varied over the study period. In this primary design, all eligible infants meeting inclusion criteria were included without sampling which improved power and minimized bias related to selection. Furthermore, it was reassuring that both the cohort and the secondary TND yielded vaccine effectiveness estimates in the same direction. Although both approaches adjusted for the same confounding factors, the effectiveness estimates from the TND were higher than those from the cohort design, which is consistent with our previous analyses of influenza vaccine effectiveness in which we also observed that the TND tended to result in higher vaccine effectiveness estimates than did our cohort analyses^24^. The TND, a case control study, has been commonly used in studies of the effectiveness of influenza vaccines and more recently COVID-19 vaccines. It is designed to better adjust for healthcare seeking behavior^25–27^, although it may also introduce other biases including selection bias^28^.The TND used analysis limited to the sample of infants who were tested for SARS-CoV-2.

The study had limitations worth noting. Vaccinations were limited only to those received during pregnancy. We did not assess whether vaccines received before pregnancy or immediately after pregnancy were associated with reduced risk of testing positive for SARS-CoV-2 in infants. The study did not adjust for maternal SARS-CoV-2 infections during pregnancy. During the study period, home testing became more prevalent. It is possible that this practice may have led to some misclassification of outcome, and we were unable to assess whether this misclassification was differential between vaccinated and unvaccinated mothers. We did not have genotyping data to confirm the variant that infected infants who tested positive and instead relied on state data regarding circulating strain predominance in the Northern California region. Like all observational studies, our study results are susceptible to residual confounding.

In conclusion, in this population-based cohort study, we found that infants born to mothers who received at least 2 doses of a mRNA COVID-19 vaccine during pregnancy were at lower risk of testing positive for SARS-CoV-2 and were at lower risk of hospitalization during the first 6 months of life compared with infants whose mothers were unvaccinated during pregnancy. Maternal vaccination was protective, but protection was lower during the Omicron period than during Delta. Protection during both periods decreased as infants aged from 2 months to 6 months. Overall, the study results support recommendations for vaccination during pregnancy to protect both mothers and their infants.

## Online Method

### Setting and Study Population

The study setting was Kaiser Permanente Northern California (KPNC), an integrated healthcare delivery organization that provides comprehensive health care to approximately 4.4 million members as of 2019. Members receive almost all their medical care at KPNC-owned facilities, including clinics, hospitals, pharmacies, and laboratories. KPNC has a comprehensive electronic health record system that captures detailed information on all medical services, including immunization, membership enrollment including place of residence, demographics, and pregnancy related care from pregnancy onset to delivery and beyond. KPNC members are similar to the broad catchment population in Northern California in terms of sociodemographic characteristics^29^. Annually, approximately 40,000 births occur at KPNC facilities.

The study was conducted among a cohort of infants born between December 15, 2020, and May 31, 2022. From this cohort, the study excluded the following infants born to: 1) mothers who were not between ages 16 and 50 years at pregnancy onset; 2) mothers who did not have a primary KPNC facility assignment; 3) mothers who were not continuous KPNC members from December 15, 2020 until delivery 4) mothers who had a positive nasal/throat swab for SARS-CoV-2 by polymerase chain reaction (PCR) prior to pregnancy onset; 5) mothers who had a positive SARS-CoV-2 antibody test documented by KPNC prior to onset of pregnancy; 6) mothers who received one or more doses of COVID-19 vaccine prior to pregnancy onset. We excluded these infants because we were primarily interested in estimating the effectiveness of at least 2 doses of mRNA vaccines received during pregnancy; 7) mothers who received other COVID-19 vaccine than mRNA vaccine during pregnancy; 8) mothers who did not receive their mRNA vaccinations in accordance with CDC recommendations – e.g., the timing between dose 1 and dose 2 was not within the recommended intervals; and 9) infants who did not become KPNC members within two calendar months of their birth. No other exclusion criteria were applied.

The KPNC Institutional review board approved and waived consent for this study.

### Outcomes

The outcomes were infant’s first positive nasal/throat swab for SARS-CoV-2 by PCR, and first COVID-19 related hospitalization, occurring during the first 6 months of life and recorded in the electronic health record.

### Exposure

The exposure of interest was mRNA COVID-19 vaccination status during pregnancy in the electronic health record. Mothers were classified as either having had ≥ 2 doses of mRNA COVID-19 vaccines during pregnancy (and completed more than 7 days prior to delivery) or not having had any COVID-19 vaccines prior to delivery. We further classified vaccination status by the trimester within which the 2^nd^ dose was received.

### Covariates

For mothers of infants in the cohort we extracted from the electronic health record data: age at pregnancy onset, race/ethnicity (Asian, Black, Hispanic, Pacific Islander, Multiracial, Native American, Other, White), the primary KPNC facility at which the woman received most of their health care, insurance payor (dichotomized as “Medicare/Medicaid/other subsidized insurance” and “Other”), neighborhood deprivation index [NDI]^30^ categorized into quartiles with higher values representing greater deprivation), pre-pregnancy body mass index (BMI=kg/m2; underweight <18.5, normal 18.5–24.9, overweight 25.0–29.9, obese ≥30.0), pre-pregnancy diabetes status, pre-pregnancy hypertension and parity (0, 1, 2, 3, ≥4). For infants, we included age, as a categorical time-changing variable in 30-day increments and preterm status defined as gestational age at birth less than 37 weeks.

### Statistical analysis

We conducted a descriptive analysis of the study population and calculated crude rates of SARS-CoV-2 infection and hospitalization by maternal vaccination status. In our primary analysis, we implemented a cohort study design where we used Cox proportional hazards models that allow for time-varying covariates to estimate the SARS-CoV-2 infection hazard ratio (HR) in infants of mothers vaccinated with at least 2 doses of mRNA COVID-19 vaccines during pregnancy versus mothers who were unvaccinated during pregnancy. We calculated vaccine effectiveness (VE) as 100% multiplied by 1 – HR. In all models, we used calendar days as the time scale to account for changes over time in SARS-CoV-2 circulation and vaccine uptake. Infants were followed from birth until first positive SARS-CoV-2 test by PCR at age 2, 4 or 6 months, with censoring due to death, health plan disenrollment, or end of follow-up (May 31, 2022). Models were adjusted for covariates listed above. To account for the correlation between infants with the same mother, we fit marginal Cox proportional hazards models using robust sandwich variance estimates. We ran separate models on the time periods associated with the Delta (7/01/2021 to 12/20/2021) and Omicron variants (12/21/2021 to 5/31/2022). We also conducted analyses based on the trimester during which the vaccine was received during pregnancy (first, second or third trimester).

We conducted secondary sensitivity analyses restricting the population to infants who received at least one SARS-CoV-2 PCR test. In this analysis, we estimated the odds ratio (OR) of vaccination of mothers of infants who tested positive for SARS-CoV-2 versus infants who tested negative using logistic regression models conditioned (stratified) on the calendar date of the test so that infants testing positive on a certain day were compared to infants testing negative on that same day. We calculated VE as 100% multiplied by 1- OR. This case-positive, control-test-negative design also referred to as the test negative design (TND) has often been used in studies of vaccine effectiveness. The TND is designed to better control for bias related to health care seeking behavior^25–27^. Models in this analysis were adjusted for the same covariates included in the primary analysis. All analyses were conducted using SAS software, v9.4. and statistical significance was assessed at two-sided p≤0.05.

## Figures and Tables

**Table 1.** Characteristics of the Study Population: Infants Born at Kaiser Permanente Northern California December 15, 2020 – May 31, 2022.

Characteristics	Infants included in the study N=30,288 n(%)	Infants whose mothers were vaccinated during pregnancy N=11,109 n(%)	Infants whose mothers were not vaccinated during pregnancy N=19,179 n(%)
Maternal age at pregnancy onset (years), mean (std)	31.62 (4.66)	32.59 (4.31)	31.06 (4.76)
Maternal age category (years)			
16 – < 25	2,092 (6.91)	393 (3.54)	1,699 (8.86)
25 – <35	20,036 (66.15)	7,047(63.44)	12,989 (67.73)
35 – <50	8,160(26.94)	3,669(33.03)	4,491(23.42)
Maternal race/ethnicity			
Asian	8,255 (27.26)	3,741 (33.68)	4,514 (23.54)
Black	1,564 (5.16)	373 (3.36)	1,191 (6.21)
Hispanic	7,405 (24.45)	2,198 (19.79)	5,207 (27.15)
Pacific Islander	246 (0.81)	74 (0.67)	172 (0.90)
Multiracial	108 (0.36)	37 (0.33)	71 (0.37)
Native American	764 (2.52)	289 (2.60)	475 (2.48)
Other/unknown/multi-racial	566 (1.87)	227 (2.04)	339 (1.77)
White	11,380 (37.57)	4,170 (37.54)	7,210 (37.59)
Parity, n (%)			
0	12,084 (39.90)	4,809 (43.29)	7,275 (37.93)
1	11,180 (36.91)	4,259 (38.34)	6,921 (36.09)
2	3,921 (12.95)	1,171 (10.54)	2,750 (14.34)
3	1,212 (4.00)	279 (2.51)	933 (4.86)
4+	576 (1.90)	130 (1.17)	446 (2.33)
Unknown	1,315 (4.34)	461 (4.15)	854 (4.45)
Medical comorbidity before pregnancy, n (%)			
Diabetes	490 (1.62)	221 (1.99)	269 (1.40)
Hypertension	2,469 (8.15)	916 (8.25)	1,553 (8.10)
Body Mass Index			
Underweight	658 (2.17)	272 (2.45)	386 (2.01)
Normal	12,478 (41.20)	4,870 (43.84)	7,608 (39.67)
Overweight	8,840 (29.19)	3,127 (28.15)	5,713 (29.79)
Obese	8,206 (27.09)	2,805 (25.25)	5,401 (28.16)
Unknown	106 (0.35)	35 (0.32)	71 (0.37)
Type of insurance			
Subsidized (Medicare/Medicaid/other subsidized insurance)	2,007 (6.63)	460 (4.14)	1,547 (8.07)
Non-subsidized	28,281(93.37)	10,649 (95.86)	17,632 (91.93)
Neighborhood Deprivation Index (quartile), n (%)			
First quartile 1 (least deprived)	7,481 (24.70)	3,651 (32.87)	3,830 (19.97)
Second quartile	8,101 (26.75)	3,010 (27.10)	5,091 (26.54)
Third quartile	7,257 (23.96)	2,379 (21.42)	4,878 (25.43)
Fourth quartile (most deprived)	7,396 (24.42)	2,046 (18.42)	5,350 (27.90)
Missing	53 (0.17)	23 (0.21)	30 (0.16)
Preterm birth	2,256 (7.45)	775 (6.98)	1,481 (7.72)
mRNA COVID-19 vaccine doses			
0 dose	19,179 (63.32)	0 (0.00)	19,179 (100)
1 dose	1,035 (3.42)	1,035 (9.32)	0 (0.00)
2 doses	9,456 (31.22)	9,456 (85.12)	0 (0.00)
3 doses	618 (2.04)	618 (5.56)	0 (0.00)
Gestational age at COVID-19 vaccination for second dose			
First trimester	2,081 (6.87)	2,081(18.73)	0 (0.00)
Second trimester	3,669 (12.11)	3,669 (33.03)	0 (0.00)
Third trimester	4,324 (14.28)	4,324 (38.92)	0 (0.00)
Gestational age at COVID-19 vaccination for those who only had one dose			
First trimester	56 (0.18)	56 (0.50)	0 (0.00)
Second trimester	46 (0.15)	46 (0.41)	0 (0.00)
Third trimester	933 (3.08)	933 (8.40)	0 (0.00)
Positive PCR test status			
Positive during first 6 months of life	940 (3.10)	391 (3.52)	549 (2.86)
Hospitalization status			
Hospitalized with positive PCR test during first 6 months of life	10 (0.03)	1 (<0.01)	9 (0.05)

**Table 2.** Effectiveness of COVID-19 Vaccination During Pregnancy Against Infant SARS-CoV-2 Infection: Cohort design

	First 2 months of life			First 4 months of life			First 6 months of life		
Infant observation period by predominant SARS-CoV-2 variant, and mother’s vaccination status	Positive test N	Crude incidence rate^1^	Adjusted^2^ VE (95% CI)	Positive test N	Crude incidence rate^1^	Adjusted^2^ VE (95% CI)	Positive test N	Crude incidence rate^1^	Adjusted^2^ VE (95% CI)
Delta period									
Unvaccinated during pregnancy	54	5.53	Reference	118	5.18	Reference	189	4.83	Reference
Received ≥2 doses during pregnancy	8	0.74	85 (67,93)	27	1.38	64 (43,78)	38	1.55	57 (36,71)
Omicron period									
Unvaccinated during pregnancy	54	17.02	Reference	138	18.13	Reference	297	20.93	Reference
Received ≥2 doses during pregnancy	54	16.18	22 (−18,48)	178	17.34	14 (−10,32)	326	16.40	12 (−4,26)

1 Rate per 100 person-years

2 Adjusted for maternal age, race/ethnicity, neighborhood deprivation index quartile, insurance payor, KPNC facility, pre-pregnancy body mass index, diabetes, hypertension, parity, child age and preterm birth status

VE= Vaccine effectiveness

**Table 3.** Effectiveness of COVID-19 Vaccination During Pregnancy Against Infant SARS-CoV-2 Infection by Infant Age at Testing, Trimester of Vaccination during Pregnancy, and by Virus Variant: Cohort design.

	First 2 months of life			First 4 months of life			First 6 months of life		
Infant observation period by predominant SARS-CoV-2 variant, and mother’s vaccination status	Positive test N	Crude incidence rate^1^	Adjusted^2^ VE (95% CI)	Positive test N	Crude incidence rate^1^	Adjusted^2^ VE (95% CI)	Positive test N	Crude incidence rate^1^	Adjusted^2^ VE (95% CI)
Delta period									
Unvaccinated during pregnancy	54	5.53	Reference	118	5.18	Reference	189	4.83	Reference
Received ≥2 doses during pregnancy									
2nd dose during 1st trimester	3	1.73	61 (−31,89)	4	1.81	48 (−54,82)	4	1.78	49 (−46,82)
2nd dose during 2nd trimester	2	0.42	91 (63,98)	11	1.39	60 (23,79)	11	1.23	65 (33,81)
2nd dose during 3rd trimester	3	0.69	87 (55,96)	12	1.28	70 (43,84)	23	1.72	54 (27,71)
Omicron period									
Unvaccinated during pregnancy	54	17.02	Reference	138	18.13	Reference	297	20.93	Reference
Received ≥2 doses during pregnancy									
2nd dose during 1st trimester	26	15.67	26 (−22,56)	58	13.18	23 (−6,44)	86	12.23	19 (−3,37)
2nd dose during 2nd trimester	12	11.26	37 (−21,67)	72	19.74	5 (−28,29)	152	19.17	3 (−19,21)
2nd dose during 3rd trimester	16	26.12	−4 (−84,41)	48	21.64	12 (−23,37)	88	17.92	18 (−4,36)

1 Rate per 100 person-years

2 Adjusted for maternal age, race/ethnicity, neighborhood deprivation index quartile, insurance payor, KPNC facility, pre-pregnancy body mass index, diabetes, hypertension, parity, child age and preterm birth status

VE=Vaccine effectiveness
